# Review of economic analysis of available interventions on idiopathic short stature: A protocol for systematic review and meta-analysis

**DOI:** 10.1097/MD.0000000000024871

**Published:** 2021-02-19

**Authors:** Boram Lee, Chan-Young Kwon

**Affiliations:** a Clinical Medicine Division, Korea Institute of Oriental Medicine, 1672 Yuseongdae-ro, Yuseong-gu, Daejeon; b Department of Oriental Neuropsychiatry, Dong-eui University College of Korean Medicine, 62 Yangjeong-ro, Busanjin-gu, Busan, Republic of Korea.

**Keywords:** cost-benefit analysis, economic evaluation, idiopathic short stature, study protocol, systematic review

## Abstract

**Background:**

Idiopathic short stature (ISS) causes a high economic burden worldwide. As part of a research project that synthesizes economic evidence for Korean medicine treatment of ISS, we describe the methods that will be used for the comprehensive review of articles that analyze health-related economic evaluation for available interventions for ISS using a systematic review methodology.

**Methods:**

Eight electronic English, Korean, and Chinese databases will be searched from their inception until December 2020 to identify studies on the economic evaluation of available interventions on ISS, without language, study design, or publication status restrictions. From the included studies, the effectiveness, utility, and cost data will be collected as the outcome measures by two researchers independently. Descriptive analysis of individual studies will be conducted. If it is judged that the interventions and outcomes of the included studies are sufficiently homogeneous, we will attempt a quantitative synthesis through meta-analysis using Review Manager version 5.4 software (Cochrane, London, UK).

**Results:**

This study will summarize the evidence regarding the economic evaluation of available interventions for ISS.

**Conclusions:**

The findings of this review will help clinicians and patients in evidence-based decision-making in clinical settings and help policy makers develop effective policies and distribute resources based on the available evidence.

## 1 Introduction

Idiopathic short stature (ISS) refers to a condition in which an individual's height is less than -2 standard deviations of the corresponding average height for the same age, sex, and population, without systemic, endocrine, nutritional, or chromosomal abnormalities that can cause growth disorders.^[1]^ ISS accounts for 80% of all patients with growth disorder,^[2]^ and is reported to have socioeconomic impacts, such as causing emotional problems due to height inferiority, especially in children and adolescents.^[3,4]^ Since the United States Food and Drug Administration approved the use of recombinant human growth hormone (GH) for ISS in 2003,^[5]^ GH has been used as a conventional medication in clinical settings. However, patient satisfaction was reported to be low due to various factors such as pain and the psychological burden caused by daily GH injections and anxiety about the side effects.^[6,7]^ In addition, there is no benefit treatment for ISS yet and the cost of GH treatment is very high worldwide, causing a high economic burden.^[8,9]^

The purpose of economic evaluation is to comparatively analyze the available actions in terms of costs and consequences, and to identify the best action based on the available evidence.^[10]^ This, along with safety and efficacy, is becoming an important area of health technology assessment. However, there is no systematic review that provides evidence regarding the health economic evaluation of available interventions for ISS.

The authors are conducting a research project that synthesizes effectiveness, safety, and economic evidence regarding Korean medicine treatment for ISS through the Korea Health Industry Development Institute, funded by the Ministry of Health & Welfare, Republic of Korea. Accordingly, a network meta-analysis was performed to compare the effect size between Korean medicine treatments of ISS. In addition, as part of this research project, we aim to comprehensively review the health-related economics analysis of all available interventions for ISS using a systematic review methodology.

## 2 Methods

### 2.1 Protocol registration

The protocol for our study has been registered on the OSF registry (URL: https://osf.io/asqj2). We reported this systematic review protocol in accordance with the Preferred Reporting Items for Systematic Review and Meta-Analysis Protocols.^[11]^

### 2.2 Ethics and dissemination

Since our study is a systematic review, ethical approval is not required as the personal information and data for individual patients will not be included. The study results will be disseminated through a manuscript in a peer-reviewed journal or presentation at a relevant conference.

### 2.3 Data sources and search strategy

The following databases will be searched by one researcher (BL) without language restrictions: Medline via PubMed, EMBASE via Elsevier, and Cochrane library. In particular, Korean databases (Oriental Medicine Advanced Searching Integrated System, Research Information Service System, and Korean Medical Database), and Chinese databases (China National Knowledge Infrastructure and Wanfang data) will be searched to collect articles on the economic evaluation of Korean medicine treatment. Each database will be searched for studies published by December 2020. To identify all eligible studies, the reference lists of relevant articles and trial registries will be searched. Not only articles published in journals but also “gray literature” including conference proceedings will be included. We will collect as many articles as possible through consultations with experts in systematic review methodology. Search terms corresponding to ISS and economic evaluation will be used as search strategies. The search strategy for Medline via PubMed is described in Table 1 and will be modified similarly for the other databases. If a manuscript describing the same research data is published in more than one journal, we will include the most comprehensively described article.

**Table 1:** Search strategies for Medline.

	Searches
#1	“idiopathic short stature”[TIAB] OR “Growth Disorders”[MH] OR “growth disorder∗”[TIAB] OR “growth retardation”[TIAB] OR “short stature”[TIAB] OR Dwarfism[MH] OR Dwarfism[TIAB] OR “Failure to Thrive”[MH] OR “failure to thrive”[TIAB] OR “growth impairment”[TIAB]
#2	“Costs and Cost Analysis”[MH] OR “Cost-Benefit Analysis”[MH] OR “Economics, Pharmaceutical”[MH] OR economic∗[TIAB] OR cost∗[TIAB] OR price∗[TIAB] OR pricing[TIAB] OR expenditure[TIAB] OR budget[TIAB] OR “Quality-Adjusted Life Years”[MH] OR “quality adjusted life year∗”[TIAB] OR QALY∗[TIAB] OR “quality adjusted life expectancy”[TIAB] OR “Markov model”[TIAB] OR “decision tree”[TIAB]
#3	#1 AND #2

### 2.4 Eligibility criteria

#### 2.4.1 Study design

We will include studies of all types and designs reporting the outcomes of interest, including randomized controlled trials, non-randomized controlled trials, review articles, and case reports. However, we will exclude articles for which only abstracts with too little information are available or the full-texts cannot be acquired.

#### 2.4.2 Participants

We will include studies targeting ISS patients with a height more than 2 standard deviations below the corresponding average height for a given age, sex, and population. We will exclude studies targeting participants with other diseases that can cause short stature such as GH deficiency. No restrictions will be imposed on the sex, age, or race of the participants.

#### 2.4.3 Interventions and comparators

The types of interventions and comparators will not be limited. Not only conventional treatment such as GH but also East Asian traditional medicine such as herbal medicine and acupuncture will be allowed.

#### 2.4.4 Outcomes

All studies in which the economic analysis of specific interventions for ISS patients was conducted will be included. The outcome measures of interests are as follows:

(1) Effectiveness data: post-treatment value or changes after treatment in growth-related anthropometric indicators, such as height, adult height, and predicted adult height, and in growth-related hormones such as GH
(2) Utility data: post-treatment value or changes after treatment in quality of life scales, such as the European Quality of Life Five Dimension scale^[12]^
(3) Cost data

Even if an economic evaluation was not performed, we will include studies that provide data on the effectiveness and cost of a particular intervention.

### 2.5 Study selection and data extraction

**Figure 1:**
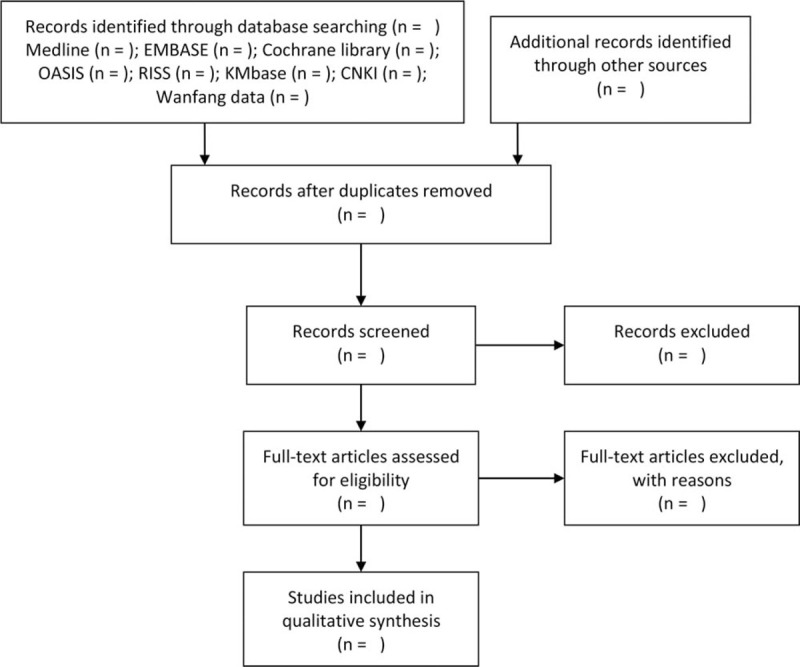
A flow diagram of the literature screening and selection processes. CNKI = China National Knowledge Infrastructure, KMbase = Korean Medical Database, OASIS = Oriental Medicine Advanced Searching Integrated System, RISS = Research Information Service System.

We will import the searched articles from all databases and sources into EndNote X8 (Clarivate Analytics, Philadelphia, USA). After removing duplicates, the titles and abstracts of the searched studies will be reviewed for the initial screening process and then the full texts of eligible studies will be reviewed for final inclusion. We will report the literature selection process according to the Preferred Reporting Items for Systematic Reviews and Meta-Analysis flow diagram (Fig. 1).

The following items will be extracted from each included study using Microsoft Office Excel 2016 (Microsoft, Redmond): the first author's name; study design; publication year; country; sample size; details about the participants and interventions; economic analysis method (including cost-minimization analysis, cost-benefit analysis, cost-effectiveness analysis, and cost-utility analysis); perspective of economic analysis; effectiveness and utility outcome and data; cost data (including direct, indirect, or both) and currency (such as euro and dollar); decision analytic model used; and main findings.

The study selection and data extraction will be conducted by two researchers (B Lee and CY Kwon) independently and disagreements between them will be resolved through discussions with another expert. If the data for the included studies are insufficient, we will contact the corresponding authors for each study via e-mail to request additional information.

### 2.6 Data analysis

Since the studies to be included are expected to be very heterogeneous with regard to the interventions, analysis methods, and so on, we will descriptively analyze individual studies. Categorical data will be presented as frequencies and percentages using Microsoft Office Excel 2016.

If it is judged that the study design, interventions, and outcomes of the included studies are sufficiently homogeneous, we will attempt a quantitative synthesis through meta-analysis using Review Manager version 5.4 software (Cochrane, London, UK). Statistical heterogeneity will be assessed using the I-squared statistic. We will consider I-squared values greater than 50% and 75% indicative of substantial and high heterogeneity. We will pool continuous outcomes using mean difference or standardized mean difference and dichotomous outcomes using risk ratio, with 95% confidence intervals. If the meta-analysis contains a sufficient number of studies, a funnel plot is used to assess publication bias.

## 3 Discussion

To the best of our knowledge, this will be the first systematic review that comprehensively summarizes the available evidence regarding the economic evaluation of available interventions for ISS. The results of this study are expected to not only help clinicians and patients in evidence-based decision-making in clinical settings, but also to help policy makers develop effective policies and distribute resources based on the available evidence. In addition, the results of this study can provide a guide, especially regarding measurement tools and analysis methods to be considered when conducting an economic evaluation of ISS treatment interventions in the future. They can also be used for the development of clinical studies with economic evaluation protocols for ISS. Especially, the results of this study will be used as basic data for a research project on the economic evaluation of Korean medicine treatment for ISS.

## Author contributions

**Conceptualization:** Boram Lee.

**Funding acquisition:** Boram Lee.

**Methodology:** Boram Lee, Chan-Young Kwon.

**Supervision:** Boram Lee.

**Writing – original draft:** Boram Lee.

**Writing – review & editing:** Chan-Young Kwon.
